# Effectiveness of Yoga and Acupuncture in Rheumatoid Arthritis: A Systematic Review and Meta-Analysis

**DOI:** 10.1155/2023/9098442

**Published:** 2023-10-06

**Authors:** Carla Andrea Cortés-Ladino, Wanderley Augusto Arias-Ortiz, Alexandra Porras-Ramírez

**Affiliations:** Universidad El Bosque, Faculty of Medicine, Research Group of Medicina Comunitaria y Salud Colectiva, Bogotá, Colombia

## Abstract

**Introduction:**

Rheumatoid arthritis is a form of inflammatory joint disease; sometimes, patients need prolonged cycles of nonsteroidal anti-inflammatory drugs and/or glucocorticoids for symptomatology management in addition to traditional disease-modifying drugs and biologics. On some occasions, doses are increased without improvement of symptoms associated with side effects; this is why, on some occasions, patients seek other types of nonpharmacological therapies, such as alternative therapies.

**Objective:**

To establish the effectiveness of alternative therapies such as yoga and acupuncture in rheumatoid arthritis by measuring disease activity with the disease activity score 28.

**Methods:**

A systematic review of the literature and meta-analysis was performed; databases such as PubMed and Embase were used to find the best available evidence of randomized clinical trials from 2017 to 2021, two researchers independently screened and extracted the necessary data, and the methodological quality and the risk of bias were assessed through the Cochrane risk-of-bias tool. The articles that applied for meta-analysis were analyzed in Jamovi version 2.2 and EPIDATA version 3.1 software.

**Results:**

Eight studies were included for qualitative analysis of which seven were included in the meta-analysis, with 550 rheumatoid arthritis patients, predominantly female. The meta-analysis evidenced a significant effect of yoga and acupuncture interventions in decreasing DAS 28 in patients with rheumatoid arthritis (four RCTs; SMD −2.51 95% CI [−2.89, −2.14], *p* ≤ 0.001, *I*^2^ 25.9%); in the yoga subgroup analysis, there was also evidence of improvement in the evaluated outcome (three RCTs; SMD −0.51 95% CI [−0.71, −0.30], *p* ≤ 0.001, *I*^2^ 0%).

**Conclusion:**

It was demonstrated that the practice of yoga and acupuncture in patients with rheumatoid arthritis helped to decrease disease activity through the improvement of pain and joint inflammation; we recommend the implementation of this type of alternative intervention associated with conventional therapies for the management of patients diagnosed with rheumatoid arthritis.

## 1. Introduction

Rheumatoid arthritis is a common form of inflammatory joint disease, which can cause progressive disability and systemic inflammation. Within 10 years of the onset, at least 50% of the affected patients are limited functionally [1]. Patients mostly require prolonged cycles of nonsteroidal anti-inflammatory drugs (NSAIDs) and glucocorticoids to manage pain, inflammation, joint stiffness, and loss of function, along with traditional and biologic disease-modifying drugs (DMARDs) [2].

In the past 10 years, important advances have been made in the pharmacological and interventional fields for the management of rheumatologic pain; however, in some cases, the doses were increased without finding any improvement of the symptoms, being considered a therapeutic failure; in addition, the side effects associated with these drugs were becoming present [3].

Due to the escalation of DMARD therapy, prolonged cycles of NSAIDs and glucocorticoids, associated with the increase of side effects and the failure of pharmacological therapies and consequently poor management of the symptomatology, patients seek other types of treatments such as alternative therapies.

Alternative therapies encompass a wide and diverse range of techniques; however, they may present side effects or no improvement of the health condition they seek to treat. In the past decade, in countries such as the United States, Canada, and the United Kingdom, the demand and use of alternative therapies have been on the rise, which has led to an increase in the regulation of these types of treatments and practices, as well as the due qualification and accreditation of their professionals [4, 5].

The World Health Organization (WHO) for the treatment of rheumatoid arthritis recommends acupuncture since it can manage autoimmune disease exerting a regulatory effect on the hemodynamic functions and changing the concentrations of neurotransmitters and neuromodulators such as endorphins, neurohumoral factors, and chemical mediators in the central nervous system [1, 2]; therefore, it sensitize peripheral and central neurons reducing and maintaining pathological pain and inflammation, having a positive effect on disease activity.

On the other hand, yoga practice focuses on the physiologically defined relaxation response and intends to balance the effect of increased uncontrolled emotions related to pain; numerous studies have examined this practice in chronic pain, finding that the main focus of the practice is on asanas (body positions), incorporating muscle strengthening and stretching combined with relaxation and breathing control. Relaxation response leads to multiple physiologic beneficial effects that enhance pain relief through reduced sympathetic activity, decreased muscular tension, modulated pain awareness, and an increased release of endogenous neuromodulators [2].

Although pharmacological therapy is the mainstay of treatment, alternative therapies also play an important role in the management of rheumatoid arthritis. Thus, it is important to establish within the diversity of nonpharmacological therapies of alternative origin, which are effective and have the best results when treating a patient, reducing pain and joint inflammation.

To achieve this, the objective of the investigation was to establish the effectiveness of alternative therapies such as yoga and acupuncture in rheumatoid arthritis by measuring disease activity with the disease activity score 28 (DAS 28).

## 2. Methodology

The protocol was registered in the International Prospective Register of Systematic Reviews (PROSPERO) database under the number CRD42022293612 [5]; the review was performed according to the Preferred Reporting Items for Systematic Reviews and Meta-Analyses (PRISMA) guidelines [6].

### 2.1. Inclusion Criteria

#### 2.1.1. Type of Studies

We included controlled clinical trials (RCTs) reported in the different meta-search engines with full text, in English, Spanish, and Chinese language, published in the last five years (2017–2021) that evaluated as the outcome, the improvement of DAS 28 in patients with rheumatoid arthritis submitted to yoga and/or acupuncture.

#### 2.1.2. Type of Participants

This review included adult patients with a diagnosis of rheumatoid arthritis, as defined by the American Academy of College of Rheumatology (ACR) classification criteria.

#### 2.1.3. Type of Interventions

We included RCTs with yoga programs and complementary therapy with acupuncture, compared with nonyoga and nonacupuncture methods plus follow-up of DMARD therapy.

#### 2.1.4. Characterization of the Outcome

It was decided to measure the average value of DAS 28 as the only outcome, after the intervention performed in each RCT (yoga or acupuncture) compared to its initial value in order to observe if the interventions had an effect on pain and inflammation improvement and, therefore, on disease activity.

### 2.2. Exclusion Criteria

No retrospective studies were considered for this systematic review, conducted in the pediatric population nor was contemplated the use of alternative therapies other than yoga or acupuncture.

### 2.3. Search Methods

Different search strategies were run, using the medical material head terms (MESH) and the Boolean operators as follows: (rheumatoid arthritis [MeSH Terms]) AND (acupuncture [MeSH Terms])) OR (yoga [MeSH Terms])) AND (DAS28)) in the meta-search engines: PubMed, Embase, Science Direct, Web of Science, Bireme, and BVS.

### 2.4. Data Collection and Analysis

#### 2.4.1. Selection of Studies

This search was reviewed and screened by two evaluators on the Rayyan platform, with 745 articles, which, after eliminating duplicates, left 741 articles, and after primary and secondary screening, eight were included in the final review.

#### 2.4.2. Data Extraction

The following data were extracted from the studies included in the review, patient characteristics (age, sex), study characteristics (study design, year of publication, country of origin, inclusion and exclusion criteria, sample size, and type of intervention performed), and study outcomes (DAS 28 value).

#### 2.4.3. Evaluation of Study Quality and Identification of Biases

Two reviewers, using the Cochrane risk-of-bias tool (RoB 2) [6], which includes the domains of the randomization process, deviation from the interventions intended to evaluate, missing outcome data, outcome measurement, selection of the reported outcome, and overall bias, assessed the methodological quality and risk of bias in the included studies independently. In case of discrepancies in the assessments, a third evaluator performed the assessment and scoring.

### 2.5. Statistical Analysis

#### 2.5.1. Measuring the Effect of the Intervention

The meta-analysis was run through JAMOVI version 2.2 [7] and EPIDAT version 3.1 [8], with the standardized difference method by means of the DAS 28 of the intervened group and control group after the intervention, associated with the number of patients treated, the deviations and standard errors of each mean of the outcome for the included articles.

The model for the estimator was the maximum likelihood model, in addition to which prediction intervals were calculated and the AIC and BIC criteria were reviewed to assess model fit.

#### 2.5.2. Heterogeneity Assessment

We calculated statistical heterogeneity between studies using the *I*^2^, Tau^2^, and the *Q* statistic with its respective *p* value; clinical heterogeneity was evaluated according to the intervention and the characteristics of the patients, and epidemiological heterogeneity was taken into account in the design of the studies.

In the case of homogeneity, fixed-effects analysis was used, and in the presence of heterogeneity, we used random-effects analysis.

#### 2.5.3. Subgroup Analyses

Subgroup analyses were planned according to the type of intervention (yoga vs. control group and acupuncture vs. control group) in the presence of low to moderate heterogeneity among clinical trials.

#### 2.5.4. Publication Bias Analysis

Publication bias was evaluated through the funnel plot and Egger regression, using JAMOVI version 2.2 [7].

## 3. Results

**Figure 1:**
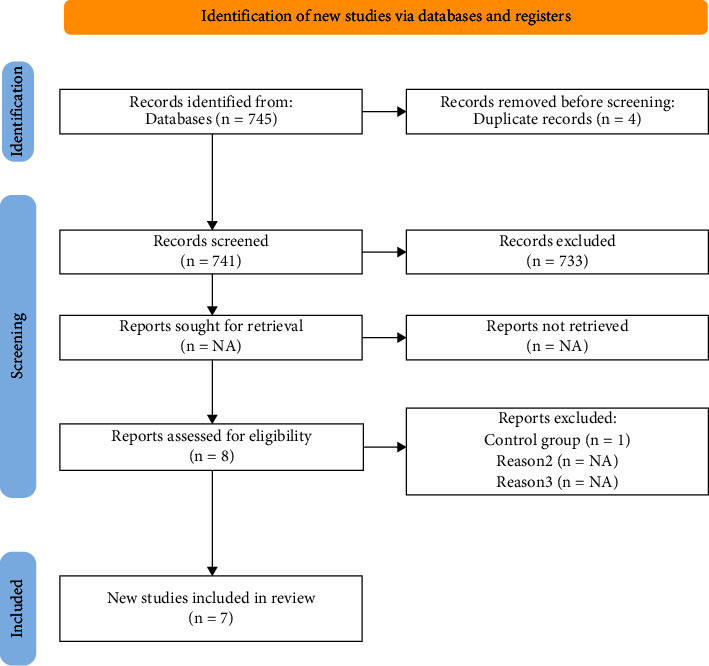
Flowchart of the selection and search process for meta-analysis. Source: adapted from Haddaway N. R., Page M. J., Pritchard C. C., & McGuinness L. A. (2022). PRISMA2020: an R package and Shiny app for producing PRISMA 2020-compliant flow diagrams, with interactivity for optimised digital transparency and open synthesis Campbell systematic reviews, 18, e1230, https://doi.org/10.1002/cl2.1230.

The initial search yielded 745 articles, of which four were duplicates, from 741 articles without duplicates, and 733 were excluded because they did not meet the inclusion criteria after reading the abstract and title. Eight articles remained for full article review, with one article excluded from the meta-analysis because the control group was healthy patients. Finally, seven RCTs with 600 patients with rheumatoid arthritis were included in the meta-analysis. The results of the literature search and the article selection and screening process are summarized in Figure 1.

### 3.1. Characteristics of the Studies

The studies were published between 2017 and 2021, of which four were conducted in India [1–3, 9], three in China [4–6], and one in Egypt [10]. The number of patients with rheumatoid arthritis enrolled in the studies ranged from 30 to 186, and their average age was between 41.3 (9.5) and 56.0 [11] years old; of the 600 patients included in the systematic review, females predominated (75.5%); finally, the average baseline DAS 28 before the intervention was between 4.77 and 8.89, and at the end of the intervention, it was between 2.43 and 4.90.

The characteristics of the eight RCTs are in Table 1.

### 3.2. Intervention Characteristics and Outcome Measurement

Regarding the interventions, for yoga, half of the studies did not specify the style practiced (50%, *n* = 2) [1, 2]; of the articles that did specify it, 25% was Patanjali's Raj yoga (*n* = 1) [3], and the other 25% was Patanjali's Ashtanga yoga (*n* = 1) [9]; the groups exposed to the intervention practiced yoga at least 30 consecutive minutes in each session with a frequency of three to five times per week, and the duration of the programs ranged from eight to twelve weeks. In these four studies [1–3, 9], the intervention group continued on RA prescription medications, as did the control group; the prescription medications were DMARDs, although it was not specified which one was prescribed. The summary of the yoga and acupuncture intervention and outcome measurement for the studies included in the qualitative analysis is in Table 2.

For acupuncture, half of the studies did not specify the type of technique practiced (50%, *n* = 2) [4, 5]; of the articles that did specify it, 25% was electroacupuncture (*n* = 1) [6], and the other 25% was laser acupuncture (*n* = 1) [10]; the groups exposed to the intervention had acupuncture sessions for at least 30 consecutive minutes, with a daily frequency (4.5) or every other day [6] or only three random days a week [10]; the duration of the programs ranged from four to twelve weeks.

In three of the four studies, the intervention group continued with formulated DMARDs [4–6], one of the four continued the same drug schedule as the control group [6]; in the control group, three of the four studies were subjected to treatment with methotrexate [4–6] associated with NSAIDs such as diclofenac [4], ibuprofen [5], and nimesulide [6], and only one of them associated gastric protection with omeprazole [6]. One of the studies included healthy patients as a control group [10].

The included randomized trials chose different primary outcomes; all assessed DAS 28 [1–6, 9, 10], six measured disease changes and inflammation through rheumatoid factor (RF), c-reactive protein (CRP) and erythrocyte sedimentation rate (ESR) [2–4, 6, 9, 10], one measured mitochondrial health [1], and one measured pre- and proinflammatory cytokine along with immunomodulatory markers [3]. Only one of the eight articles observed symptoms and/or signs of blood stasis and assessed active symptomatology by qualitative measurement [5], and finally, only one measured communicative markers of body and mind, associated with the assessment of changes in quality of life [9], as a minor outcome.

### 3.3. Methodological Assessment and Risk of Bias

In the RCTs classified as “intention to treat” [1, 3–6, 9, 10], two did not adequately explain the process of randomization of participants, nor did they adequately explain the measurement of outcomes [4, 10], three of the seven had deviation from the interventions intended to measure [4, 9, 10], all had low risk of bias in terms of reporting missing data in the outcomes, and the outcomes reported (domains three and five); the only RCT classified as “per protocol” [2] had low risk of bias in all five domains assessed. On average, five of the eight studies were rated as “low risk” [1–3, 5, 6], while the other three as “some doubt” [4, 9, 10]. The risk-of-bias description of each study is given in Table 3.

### 3.4. Evaluation of Effect Size

**Figure 2:**
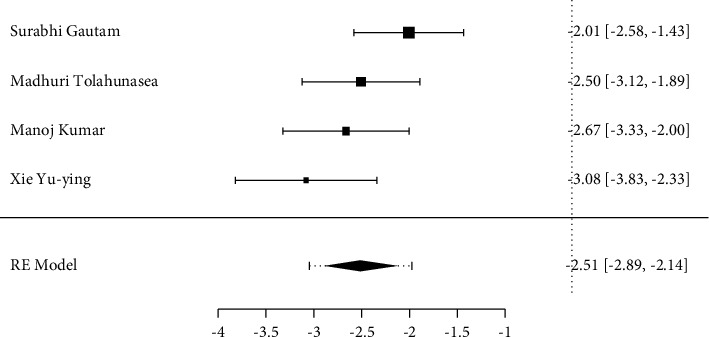
Meta-analysis of the DAS 28.

The analysis on disease activity was performed through the measurement of DAS 28 before and after the implementation of each intervention, using mean differences as a standardized measure, through the random-effects model; for the final analysis, of all seven studies, the four that remained were the four that showed homogeneity in the model [1, 3, 6, 9], demonstrating a standardized mean difference = −2.51 (95% CI [−2.89, −2.14]) and *p* value ≤0.001), a nonsignificant heterogeneity of the results (*Q* = 5.416, *p* = 0.1438, tau^2^ = 0.0380, *I*^2^ = 25.97%), and a maximum likelihood model analysis through the information criteria AIC = 7.700 and BIC = 6.473 evidencing model fit from the regression (Figure 2).

**Figure 3:**
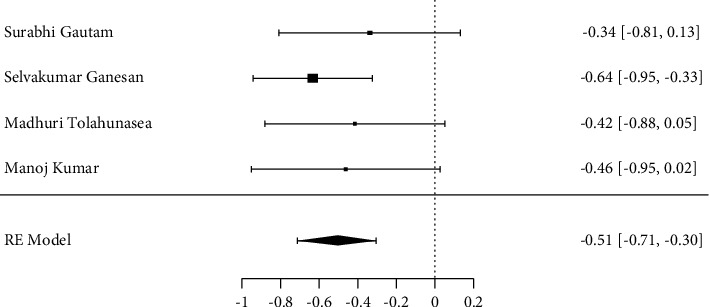
Meta-analysis of the DAS 28 in the yoga group.

For the analysis of subgroups, taking into account the high heterogeneity between acupuncture, meta-analysis was not performed. For yoga, a standardized mean difference = −0.51 (95% CI [−0.71, −0.30]) and a *p* value ≤0.001, homogeneity of the results (*Q* = 1.336, *p* = 0.7205, tau^2^ = 0.00, *I*^2^ = 0.0%), and maximum likelihood model analysis through the information criteria AIC = 0.519 and BIC = −0.709 evidencing model fit from regression (Figure 3).

### 3.5. Sensitivity Analysis

Through fixed-effects analysis, the sensitivity analysis for the four RCTs included in the meta-analysis showed that the standardized mean differences ranged from −2.01 (95% CI [−2.58, −1.43]) to −3.08 (95% CI [−3.83, −2.33]) with an overall effect of −2.51 (95% CI [−2.89, −2.14]); for subgroup yoga, the sensitivity analysis was also performed through fixed effects, and the standardized mean difference ranged from −0.34 (95% CI [−0.81, 0.13]) to −0.64 (95% CI [−0.95, −0.33]) with an overall effect at −0.51 (95% CI [−0.71, −0.30]).

### 3.6. Publication Bias

**Figure 4:**
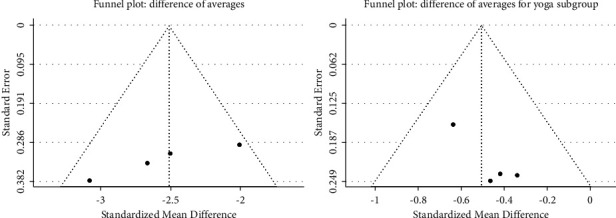
Funnel plot of difference of averages.

Egger's test for the overall meta-analysis showed asymmetry with a value of *p* = 0.025; however, when reviewing the rank correlation test, no asymmetry was found with a value of *p* = 0.083; for the yoga subgroup, Egger's test showed no asymmetry with a value of *p* = 0.283. The funnel plot of the mean differences is evident in Figure 4.

### 3.7. Security

None of the included RCTs reported adverse effects.

## 4. Discussion

### 4.1. Summary of Evidence

This systematic review summarized the results of eight randomized clinical trials on the efficacy of yoga and acupuncture compared to no intervention in the control group in patients with RA, and seven of these eight RCTs entered the meta-analysis, evaluating the effect of these therapies on disease activity.

Overall, the results of the meta-analysis suggest that practicing yoga and performing acupuncture are beneficial since they improve disease activity, decreasing DAS 28 by −2.51 points [95% CI −2.89 to −2.14]; when performing the analysis by subgroups, it was only possible to review the group that underwent yoga (1–3.6) since the acupuncture studies presented high heterogeneity, in which it was evidenced that yoga alone slightly improves disease activity, decreasing DAS 28 by −0.51 points [95% CI −0.71, −0.30].

When evaluating the clinical trials that studied the effect of acupuncture [4–6], a significant difference between the average ages of the intervened group is evident, associated with exposure times that ranged from 30 days to three months. However, these results show that yoga and acupuncture performed over a period of eight to twelve weeks decrease disease activity and thus inflammation and joint pain, without discontinuing conventional drug therapy.

### 4.2. Comparison with Previous Revisions

In the last five years, only one systematic review with meta-analysis has been performed on the benefit of yoga in RA, finding that yoga practice improves physical function through HAQ-DI, disease activity through DAS 28, and grip strength compared to control groups not exposed to the intervention; however, the recommendation was weak given methodological limitations.

Regarding the study of acupuncture, more studies published since 2017 were found; one of them performed a comparison of acupuncture techniques for the treatment of arthritis finding that electroacupuncture combined with conventional DMARDs improved DAS 28, while firewater therapy combined with DMARDs improved pain and decreased serological markers [12], and other studies concluded that acupuncture techniques in combination with Western medicine benefit the clinical condition in RA, having a repercussion in clinical markers and improving clinical symptoms to a significant degree [11, 13].

In the last reviewed article, Chou et al. concluded that acupuncture alone or combined with other treatment modalities is beneficial for arthritis clinical conditions, with no reported adverse effects, and improvement of functionality and quality of life, through mechanisms such as anti-inflammatory, antioxidant, and immune system regulating effect; however, they found inconsistencies about clinical effectiveness and lack of clinical trials with better methodological designs [14].

### 4.3. Limitations and Quality of Evidence

First, although the number of clinical studies conducted in the area of alternative therapies has increased, their number is still scarce compared to conventional therapies, so finding evidence from the last five years was difficult. Second, when assessing the methodological quality and risk of bias, 30% of the studies presented doubts in the overall assessment, concentrating on the process of randomization of patients and deviations of the intervention intended to measure the outcome. Third, the RCTs varied in terms of the age of the population, the number of patients included in the study, and the time of exposure to the intervention; this generated heterogeneity and, therefore, greater difficulty in the interpretation of the results; for example, thus, to improve heterogeneity between acupuncture studies, meta-analysis was not performed.

### 4.4. Implications for Clinical Practice

This meta-analysis suggests that practicing yoga and performing acupuncture help to improve disease activity in RA patients, finding in the analysis, a standardized mean difference of −2.51 (95% CI [−2.89, −2.14]), and *p* value ≤0.001); therefore, it is recommended to use them as complementary nonpharmacological therapies in the treatment of the disease, given that they are low-risk and beneficial interventions. Future meta-analyses should be performed to continue generating evidence of quality and impact.

### 4.5. Implications for Future Studies

Clinical trials with a more robust methodology and, therefore, of better quality should be conducted through the reporting of RCTs following a specific methodology such as CONSORT (Consolidated Standards of Reporting Trials) [15] to ensure methodological quality, guarantee and describe an adequate randomization and blinding process to avoid selection bias, and finally, conduct studies with a larger sample size, guaranteeing a bank of interested patients registered for future clinical trials.

### 4.6. Are Yoga and Acupuncture Effective in Treating Rheumatoid Arthritis?

Currently, the cause of rheumatoid arthritis remains to be established, so the goal of treatment is to ensure the patient's quality of life through adequate management of symptoms, prevention of joint damage, prevention of joint deformity, and ensuring functionality; the American Academy of Rheumatology (ACR) has established that nonpharmacological interventions such as stretching, strengthening exercises, and physical conditioning can be beneficial for RA [16].

The practice of yoga involves specific body postures (asanas), concentration practice (dharana), meditation (dhyana), and breathing regulation (pranayamas) [17]; this practice specializes in breathing and relaxation helping to reduce chronic pain; the postures have been found to stimulate the parasympathetic nervous system and therefore help to reduce the cycle of pressure and pain, reducing pain of chronic origin; additionally, including a variety of postures helps to strengthen the muscular system and improving the functionality of patients. We found a statistically significant standardized mean difference for yoga (−0.51 (95% CI [−0.71, −0.30]) and a *p* value ≤0.001).

On the other hand, acupuncture involves moxibustion, electroacupuncture, hot needle, and fire needle therapy [12]; it has been recognized that this therapeutic technique improves symptoms, delays the progression of the disease, and reduces pain in patients [11].

Finally, considering the role of inflammation in RA and the significant effect found of these therapies after a period of 8 to 12 weeks on DAS 28, the main objective was met; however, the clinical trials studied presented certain limitations, limiting the meta-analysis possibly associated with small sample sizes.

## 5. Conclusions

For this systematic review and meta-analysis, it was demonstrated that the practice of yoga and acupuncture in patients with rheumatoid arthritis helped to decrease disease activity through the improvement of pain and joint inflammation, compared to control groups in which these interventions were not performed and conventional pharmacological therapies were continued.

From this point of view, we recommend the implementation of this type of alternative intervention associated with conventional therapies for the management of patients diagnosed with rheumatoid arthritis. However, these conclusions are limited by methodological considerations such as small sample sizes, so clinical trials with larger populations, longer intervention times, high methodological quality, and reproducibility should be conducted.

## Figures and Tables

**Table 1:** Characteristics of the studies.

Study	Country	Intervention	Control group	Sample size (*n*)	Patients included		Average age		Male : female ratio		Average DAS 28 before the intervention		Average DAS 28 after the intervention	
					Intervention	Control	Intervention (±*S*)	Control (±*S*)	Intervention	Control	Intervention (±*S*)	Control (±*S*)	Intervention (±*S*)	Control (±*S*)
(1) Surabhi Gautam	India	Yoga	FARME	70	35	35	45.6 (7.9)	44.5 (7.7)	H : 6; M : 29	H : 8; M : 27	5.20	5.10	4.50	4.80
(2) Selvakumar Ganesan	India	Yoga	FARME	186	83	83	41.3 (9.5)	42.5 (7.1)	H : 5; M : 63	H : 7; M : 68	4.95	4.77	2.99	3.49
(3) Madhuri Tolahunasea	India	Yoga	FARME	72	36	36	45.7 (1.6)	42.1 (1.7)	H : 7; M : 29	H : 9; M : 27	5.13	4.95	4.42	4.80
(4) Wen-zhong Cao	China	Acupuncture	FARME + AINE	60	30	30	56.0 (14)	54.0 (11)	H : 6; M : 25	H : 5; M : 24	8.89	8.14	3.01	4.12
(5) Zhu Yan	China	Acupuncture	FARME + AINE	56	28	28	51.0 (13)	53.0 (13)	H : 7; M : 21	H : 7; M : 21	8.68	8.59	2.45	3.16
(6) Manoj Kumar	India	Yoga	FARME	66	33	33	45.1 (8.7)	43.9 (9.3)	H : 5; M : 28	H : 8; M : 25	5.20	5.00	4.50	4.90
(7) Xie Yu-ying	China	Acupuncture (electro)	FARME + AINE	60	30	30	51.8 (13.1)	52.1 (13.3)	H : 8; M : 22	H : 7; M : 23	8.62	8.49	2.43	3.05
(8) Atef M	Egypt	Acupuncture (laser)	Healthy patients	50	30	29	43.6 (11.3)	33.1 (11.1)	H : 4; M : 26	H : 10; M : 10	5.80	N/A	3.60	N/A

DMARDs, disease-modifying drugs; NSAIDs, nonsteroidal anti-inflammatory drugs; DAS 28, disease activity score 28.

**Table 2:** Interventions and outcome measures.

No.	Study	Type of intervention	Duration, frequency, and duration of intervention		Evaluated results
			Intervention	Control	
1	Surabhi Gautam	Yoga (NE)	8 weeks of yoga practice, 120 minute sessions, plus FARME	Continuation of formulated FARMEs	Measurement of changes in disease activity through DAS 28. Assessment of mitochondrial health status. Measurement of functional changes
2	Selvakumar Ganesan	Yoga (NE)	12 weeks of yoga practice, 30-minute sessions 3 times a week, plus FARME	Continuation of formulated FARMEs	Measurement of disease activity through DAS 28, inflammatory markers, and heart rate variability
3	Madhuri Tolahunasea	Yoga (Patanjali's Raj yoga)	8 weeks of yoga practice, 120 minute sessions 5 times per week, plus FARME	Continuation of formulated FARMEs	Assess change in RA severity through DAS 28, inflammatory markers, pro- and anti-inflammatory cytokines, and immunomodulatory markers
4	Wen-zhong Cao	Acupuncture (NE)	Triple therapy administered every 3 days consecutively for 10 times for 30 days, plus DMARDs	Methotrexate 10 mg weekly, diclofenac 0.3 gr dose, folic acid 5 mg weekly for 30 days	Measurement of changes in disease activity through RF, DAS 28, CRP, and ESR
5	Zhu Yan	Acupuncture (NE)	Basic points stimulated were Ganshu, Shenshu, Hegu, Quchi, and Zusanli, administered every day for 6 consecutive days a week for 3 courses, associated with FARME	Methotrexate 10 mg weekly, ibuprofen 0.3 g twice a day, folic acid 5 mg weekly; one course of treatment equivalent to 30 days, and 3 courses were necessary	Measurement of serological indices, changes in disease activity through DAS 28, observation of signs and/or symptoms of blood stasis, and assessment of active symptomatology through qualitative measurement
6	Manoj Kumar	Yoga (Patanjali's Ashtanga)	8 weeks of practice with Patanjali's Ashtanga yoga component, 120-minute sessions 5 times a week, plus FARME	Continuation of formulated FARMEs	Measurement of changes in disease activity through DAS 28, inflammatory markers, mind-body communication markers, and assess changes in quality of life
7	Xie Yu-ying	Acupuncture (electro)	1 session every 2 days for 12 weeks, plus formulated DMARDs + NSAIDs (control group scheme)	Methotrexate 10–12.5 mg weekly, nimesulide 0.1 mg twice a day, omeprazole 100 mg 3 times a day depending on the patient	Assessment of active symptomatology through qualitative measurement, DAS 28, ESR, CRP, and evaluation of efficacy
8	Atef M	Acupuncture (laser)	Laser acupuncture (904 nm, output power 100 mW, irradiation time 1 minute), 3 days a week for 4 weeks	No intervention, healthy patients	Measurement of disease activity through DAS 28, RF, inflammation markers, oxidation markers, antioxidant enzymes, and ATP levels

NE, not specified; DMARDs, disease-modifying drugs; NSAID, nonsteroidal anti-inflammatory drug; DAS 28, disease activity score 28; RF, rheumatoid factor; CRP, c-reactive protein; ESR, erythrocyte sedimentation rate; ATP, adenosine triphosphate.

**Table 3:** Bias risk assessment.

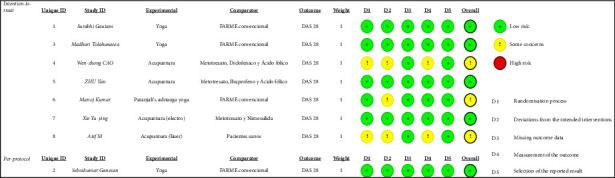

Low risk, low risk of bias; some concerns, some doubts; high risk, high risk of bias; DAS 28, disease activity score 28; DMARDs, disease-modifying drugs. Low risk, some concerns, high risk, D1 randomisation process, D2 deviations from the intendeds interventions, D3 missing outcome data, D4 measurement of the outcome, and D5 selection of the reported result.

## Data Availability

The data used to support the findings of this study may be released upon application to the authors, who can be contacted at cacortesl@unbosque.edu.co.

## References

[1] Adly A. S., Adly A. S., Adly M. S. (2022). Effects of laser acupuncture tele-therapy for rheumatoid arthritis elderly patients. *Lasers in Medical Science*.

[2] Chen L., Michalsen A. (2017). Management of chronic pain using complementary and integrative medicine. *BMJ*.

[3] Novella-Navarro M., Plasencia C., Tornero C. (2020). Clinical predictors of multiple failure to biological therapy in patients with rheumatoid arthritis. *Arthritis Research and Therapy*.

[4] Ferrer Acosta P. A., Higuera Rodríguez A. K. (2021). Factors associated with therapeutic failure of anti-TNF biologic drugs in patients with rheumatoid arthritis in the IPS Biomab. https://repository.urosario.edu.co/handle/10336/30810.

[5] Lopera Pareja E. H. (2019). EL debate político sobre las terapias alternativas Y complementarias en españa en la interfaz entre ciencia, política Y sociedad (1979-2018). *Perspectivas de la comunicación*.

[6] Page M. J., McKenzie J. E., Bossuyt P. M. (2021). The PRISMA 2020 statement: an updated guideline for reporting systematic reviews. *BMJ*.

[7] The jamovi project (2022). Jamovi (Version 2.2). https://www.jamovi.org/about.html.

[8] Hervada Vidal X., Santiago Pérez M., Vázquez Fernández E., Castillo Salgado C., Loyola Elizondo Luis Carlos Silva Ayçaguer E. (2006). Epidat 3.0 program for epidemiological analysis of tabulated data. https://www.sergas.es/Saude-publica/Documents/1938/General20Help.pdf.

[9] Cortes C., Arias W., Porras A. (2022). Alternative therapies in rheumatoid arthritis. A systematic review. https://www.crd.york.ac.uk/prospero/display_record.php?ID=CRD42022293612.

[10] Higgins J. P., Savović J., Page M. J., Elbers R. G., Sterne J. A. (2022). Chapter 8: assessing risk of bias in a randomized trial [Internet]. https://training.cochrane.org/handbook/current/chapter-08.

[11] Lu H. L., Chang C. M., Hsieh P. C., Wang J. C., Kung Y. Y. (2022). The effects of acupuncture and related techniques on patients with rheumatoid arthritis: a systematic review and meta-analysis. *Journal of the Chinese Medical Association*.

[12] Ye X., Chen Z., Shen Z., Chen G., Xu X. (2020). Yoga for treating rheumatoid arthritis: a systematic review and meta- analysis. *Frontiers of Medicine*.

[13] Wan R., Fan Y., Zhao A. (2022). Comparison of efficacy of acupuncture-related therapy in the treatment of rheumatoid arthritis: a network meta-analysis of randomized controlled trials. *Frontiers in Immunology*.

[14] Chou P. C., Chu H. Y. (2018). Clinical efficacy of acupuncture on rheumatoid arthritis and associated mechanisms: a systemic review. *Evidence-based Complementary and Alternative Medicine*.

[15] Schulz K. F., Altman D. G., Moher D. (2010). CONSORT 2010 Statement: updated guidelines for reporting parallel group randomised trials. *BMJ*.

[16] Rahnama N., Mazloum V. (2012). Effects of strengthening and aerobic exercises on pain severity and function in patients with knee rheumatoid arthritis. *International Journal of Preventive Medicine*.

[17] Varambally S., George S., Gangadhar B. N. (2020). Yoga for psychiatric disorders: from fad to evidence-based intervention?. *The British Journal of Psychiatry*.

